# Genomic selection and complex trait prediction using a fast EM algorithm applied to genome-wide markers

**DOI:** 10.1186/1471-2105-11-529

**Published:** 2010-10-22

**Authors:** Ross K Shepherd, Theo HE Meuwissen, John A Woolliams

**Affiliations:** 1 School of Information and Communication Technology, CQUniversity, Rockhampton 4702, Australia; 2 Institute Animal and Aquacultural Sciences, Norwegian University of Life Sciences, Box 5003, N1432 As, Norway; 3 Genetics and Genomics, The Roslin Institute and Royal (Dick) School of Veterinary Studies, University of Edinburgh, Roslin, Midlothian EH25 9PS, UK

## Abstract

**Background:**

The information provided by dense genome-wide markers using high throughput technology is of considerable potential in human disease studies and livestock breeding programs. Genome-wide association studies relate individual single nucleotide polymorphisms (SNP) from dense SNP panels to individual measurements of complex traits, with the underlying assumption being that any association is caused by linkage disequilibrium (LD) between SNP and quantitative trait loci (QTL) affecting the trait. Often SNP are in genomic regions of no trait variation. Whole genome Bayesian models are an effective way of incorporating this and other important prior information into modelling. However a full Bayesian analysis is often not feasible due to the large computational time involved.

**Results:**

This article proposes an expectation-maximization (EM) algorithm called emBayesB which allows only a proportion of SNP to be in LD with QTL and incorporates prior information about the distribution of SNP effects. The posterior probability of being in LD with at least one QTL is calculated for each SNP along with estimates of the hyperparameters for the mixture prior. A simulated example of genomic selection from an international workshop is used to demonstrate the features of the EM algorithm. The accuracy of prediction is comparable to a full Bayesian analysis but the EM algorithm is considerably faster. The EM algorithm was accurate in locating QTL which explained more than 1% of the total genetic variation. A computational algorithm for very large SNP panels is described.

**Conclusions:**

emBayesB is a fast and accurate EM algorithm for implementing genomic selection and predicting complex traits by mapping QTL in genome-wide dense SNP marker data. Its accuracy is similar to Bayesian methods but it takes only a fraction of the time.

## Background

Genome-wide association (GWA) studies are being used more often for risk prediction in humans and trait prediction in livestock. Such studies associate individual single nucleotide polymorphisms (SNP) from a dense genome-wide panel with between-individual variation in traits. The GWA provides measures of strength of association and estimates of the size of the effect of each SNP even though SNP identified as being of predictive value are unlikely to be causative. These GWA studies have had limited success as the individual effects of loci are often small and relatively few loci pass the very stringent statistical testing criteria imposed. The detected variants can be used to construct genetic profiles [1,2] but jointly the loci identified often explain less than 10% of the phenotypic variance [2-4]. This small fraction of variance explained is due in part to the stringent statistical thresholds required for identification in GWA studies [5]. Nevertheless the scope of the genomic information provided by high throughput technology using dense SNP panels remains of considerable potential.

Researchers in other fields, in particular animal and plant breeding, have developed methods of prediction of genetic value that use all available marker information simultaneously and do not apply such stringent tests of statistical significance [6,7]. Thus, instead of testing hundreds of thousands of separate hypotheses of 'is this single SNP associated with the trait' as in GWA, the problem is modified to 'what function of the entire SNP information provides the best predictor of the trait'. The outcome of these approaches is that many more loci are used in prediction. Although the set will now include false positive loci it also includes many more true positive effects and the overall predictive power is much improved [8]. This approach to genome-wide prediction is called genomic selection and is being applied to livestock in practice [9].

Different statistical approaches to genomic selection have been attempted. One approach is to use the markers to construct the realised relationship matrix, rather than an expected one based upon pedigree, followed by use of this realised relationship matrix in established BLUP procedures [8]. When BLUP is used for genomic selection (hereafter called GS-BLUP) the prior distribution of the marker effects is assumed normal, with the variance of the prior distribution being equal for each marker. But this "equal variance" assumption is biologically unrealistic as many markers will lie in regions that are not involved in trait determination and so contribute no trait variance. This was the finding in [6] where simulations of genomic selection found that GS-BLUP was less accurate than Bayesian methods which allowed marker specific variances which cause differential shrinkage of marker effects.

Differential shrinkage of marker effects across the genome can be performed by assuming the marker effects are normally distributed with variances which are independent random variables following a specific distribution. BayesA [6] assumes marker variances follow an inverted chi-square distribution while Bayesian LASSO (BayesL) [10] assumes an exponential distribution. Integrating out the variances it can be shown that the conditional distribution for the marker effects is a double-exponential (DE) for BayesL and a *t*-distribution for BayesA. As the DE places more density at zero than a *t*-distribution this suggests that more shrinkage will occur for small effects with BayesL than with BayesA. In fact the original LASSO [11] can be interpreted as a Bayesian posterior mode when an independent DE prior is assigned to each marker effect as shown in equation (2) in [10]. However with dense marker data, many SNP will not contribute to predicting QTL genotypes through LD and the LASSO may not perform enough shrinkage of small marker effects to comply with this prior knowledge [12]. A somewhat similar conclusion was demonstrated in [6] for the *t*-distribution prior by comparing two Bayesian methods called BayesA and BayesB. BayesB used a prior mixture which assumed a BayesA prior for a small proportion of markers and allowed the rest of the marker effects to be precisely zero *a priori*. BayesB was shown to increase selection accuracy in simulated data when compared to BayesA. However this comparison has not been conducted in a full Bayesian analysis using a DE prior like in BayesL.

A major problem associated with a full Bayesian analysis is the computing time required to fit the model. The challenge is to fit hundreds of thousands of SNP to many thousands of individuals with genotypes. Markov Chain Monte Carlo (MCMC) techniques such as Gibbs sampling are tractable when the dimensionality and data size are small. However this is not the case with dense SNP data and thus has led to the development of fast algorithms for Bayesian-like marker selection models, involving either heuristic approximations to fit into standard BLUP models [9] or an iterated conditional expectation (ICE) approach [13] which iterates an analytical calculation of each SNP's conditional posterior mean. However it is unclear in what sense the solutions of these fast algorithms are optimal.

Expectation maximization (EM) algorithms can use the information in a prior distribution through the calculation of a maximum *a posteriori *(MAP) estimate [14] and are usually much faster than a full Bayesian approach. This result was demonstrated in an EM algorithm developed for implementing genomic selection [15]. In this paper we suggest a different formulation of the SNP prior mixture compared to the EM algorithm called wBST which was developed in [15]. This results in a number of advantages which will be discussed later. Hence this paper investigates a solution to the Bayesian SNP selection model through an EM algorithm which has a solid statistical foundation compared with the fast heuristic approaches. In the sections that follow (i) we develop an algorithm (called emBayesB) using standard EM theory, (ii) we propose an implementation to work with the dimensionality that is encountered in human data sets, (iii) we benchmark emBayesB by analysing a simulated workshop data set, and finally (iv) we explore the shrinkage features of emBayesB both analytically and graphically.

## Methods

### Data model for SNP effects

Each of the *n *individuals in the study is genotyped for *m *SNP markers and has a record for a continuous trait *y*. The trait is assumed to depend on alleles of unknown QTL which, either directly or indirectly through LD, induce an association with the SNP markers. We assume that SNP marker *j *has two alleles, 0 and 1, with 1 being the reference allele which has a frequency *p*_*j *_in the *n *individuals. The three possible genotypes '0_0', '0_1' and '1_1' for SNP marker *j *are coded 0, 1 and 2 respectively, and are standardised by subtracting the mean (*2p*_*j*_) and dividing by the standard deviation (2pj(1-pj))12 to produce the *n *× *1 *vector of standardised frequencies **b**_*j *_which satisfies the identities **1**'**b**_*j *_= *0 *and **b**_*j*_'**b**_*j *_= *n *due to the standardisation which simplifies the algebra.

As each of the *n *individuals is genotyped for *m *SNP markers we can construct an *n *× *m *standardised frequency matrix **B **consisting of the *m *column vectors **b**_*j*_. We assume a linear model for the 'SNP mediated' effects of the QTL, namely **y **= **Bg + e **where **y **is the *n × 1 *vector of phenotypic records, **g **is the *m × 1 *vector of SNP effects and **e **is an *n × 1 *vector of residuals which are assumed independent and identically distributed normal random variables i.e. $\text{e}\sim N\left(0,\;\text{I}\sigma_{e}^{2}\right)$. Hence $\text{y}|\text{g}\sim N\left(\text{B}g,\;\text{I}\sigma_{e}^{2}\right)$.

### Missing data and SNP prior distribution

We assume *a priori *that a proportion *γ *of the SNP markers are in LD with at least one QTL and that an unknown binary variable *z*_*j *_(the missing data) indicates whether SNP *j *is in LD with QTL. That is, *a priori*

(1) $$p(z_{j})=\{\begin{array}{c} \;\;\gamma \;\;\text{for}\;z_{j}=1\\ 1-\gamma \;\;\text{for}\;z_{j}=0 \end{array}$$

If *z*_*j *_= *1 *(i.e. SNP *j *is in LD with QTL), the SNP effect *g*_*j *_is assumed to be from a DE distribution with parameter *λ *i.e. $h\left(g_{j}\right)=\frac{1}{2}\lambda \;\text{exp}\left(-\lambda \left|g_{j}\right|\right)$ where |*x*| is the absolute value of *x*. If *z*_*j *_= *0 *(i.e. SNP *j *is not in LD with QTL), the SNP effect *g*_*j *_is assumed to be from a Dirac Delta (DD) distribution which has all its probability mass at zero i.e. *δ*(*g*_*j*_) if *g*_*j *_≠ *0 *such that $\int_{a}^{b}\delta \left(g\right)\;dg=1$ where *a *<*0 *<*b*. Hence the conditional distribution of *g*_*j *_given *z*_*j *_is

(2) $$p(g_{j}\;|z_{j})=\{\begin{array}{c} \frac{1}{2}\lambda \;\text{exp}\left(-\lambda \left|g_{j}\right|\right)\;\;\text{for}\;z_{j}=1\\ \delta (g_{j})\;\;\;\;\;\;\text{for}\;z_{j}=0 \end{array}$$

Now the joint prior *p*(*z*_*j*_, *g*_*j*_) is as follows

(3) $$p(z_{j},\;g_{j})=p(z_{j})p(g_{j}|\;\;z_{j})={\left(\frac{1}{2}\gamma \lambda \;\text{exp}\left(-\lambda \left|g_{j}\right|\right)\right)}^{z_{j}}{\left(\left(1-\gamma )\;\delta (g_{j}\right)\right)}^{1-z_{j}}$$

Assuming independence of the *m *SNP effects, the joint prior for **z **and **g **is

(4) $$p(\text{z},\;\text{g})=\prod_{j=1}^{m}p(z_{j})p(g_{j}|\;z_{j})=\prod_{j=1}^{m}{\left(\frac{1}{2}\gamma \lambda \;\text{exp}\left(-\lambda \left|g_{j}\right|\right)\right)}^{z_{j}}{\left(\left(1-\gamma )\;\delta (g_{j}\right)\right)}^{\left(1-z_{j}\right)}$$

### Posterior distribution and EM algorithm

Apart from a normalising constant, the posterior distribution *p*(**z, g|y**) is

(5) $$p\left(\text{z},\;\text{g}|\text{y}\right)=\frac{p\left(\text{z},\;\text{g}\right)\;f\left(\text{y}|\text{z},\;\text{g}\right)}{p\left(\text{y}\right)}\propto \prod_{j=1}^{m}{\left(\frac{1}{2}\gamma \lambda \;\text{exp}\left(-\lambda \left|g_{j}\right|\right)\right)}^{z_{j}}{\left(\left(1-\gamma )\;\delta (g_{j}\right)\right)}^{\left(1-z_{j}\right)}f\left(\text{y}|\;\text{g}\right)$$

where $f\left(\text{y}|\;\text{g}\right)=\prod_{i=1}^{n}\frac{1}{{\left(2\pi \right)}^{0.5}\sigma_{e}}\text{exp}\left[-\frac{1}{2\sigma_{e}^{2}}{\left(y_{i}-\sum_{j=1}^{m}b_{ij}\;g_{j}\right)}^{2}\right]$ is the data likelihood. Taking logarithms we can show that the log posterior is proportional to the following

(6) $$\begin{array}{l} \log p\left(\text{z},\;\text{g}|\text{y}\right)\;\;\propto \;\;\sum_{j=1}^{m}z_{j}\log\left(\frac{1}{2}\gamma \lambda \right)+\sum_{j=1}^{m}\left(1-z_{j}\right)\log\left(1-\gamma \right)-\lambda \sum_{j=1}^{m}z_{j}\left|g_{j}\right|\\ \;\;\;\;\;\;\;+\sum_{j=1}^{m}\left(1-z_{j}\right)\log\left(\delta \left(g_{j}\right)\right)-\frac{n}{2}\log\sigma_{e}^{2}-\frac{1}{2\sigma_{e}^{2}}{\sum_{i=1}^{n}\left(y_{i}-\sum_{j=1}^{m}b_{ij}\;g_{j}\right)}^{2} \end{array}$$

To maximize the log posterior, we use *z*_*j *_as missing data in an EM algorithm [14]. In the E-step we evaluate $E_{z}\left[\log p\left(\text{z}|{\overset{\wedge }{\text{g}}}^{k},\text{y}\right)\right]$. As log *p*(**z**, **g**|**y**) is linear in *z*_*j*_, we only need to calculate $E\left[z_{j}|{\overset{\wedge }{\text{g}}}^{k},\text{y}\right]$ at each E-step where ${\overset{\wedge }{\text{g}}}^{k}$ is the vector of SNP estimates at iteration *k*. Additional file 1 derives an analytical expression for $\gamma_{j}^{k}\left(=E\left[z_{j}|{\overset{\wedge }{\text{g}}}^{k},\text{y}\right]\right)$ which is the posterior probability that SNP *j *is in LD with at least one QTL given the data and the current estimates at iteration *k*. For the M-step, we fix $\gamma_{j}^{k}$ and maximize *E*_*z*_[log *p*(**z**, **g**|**y**)] with respect to the parameters *g*_*j*_, *γ*, *λ *and $\sigma_{e}^{2}$.

### Estimators of *g_j_, γ, λ *and $\sigma_{e}^{2}$ for the M-step

In Additional file 2, it is shown that the maximum *a posteriori *(MAP) estimate of ***g***_*j *_is

(7) $${\overset{\wedge }{g}}_{j}=\{\begin{array}{c} \max\left[0,\;\;\gamma_{j}^{k}\left(G_{j}-\overset{\wedge }{\lambda }{\overset{\wedge }{\sigma }}^{2}\right)\right]=\max\left[0,\;\;\gamma_{j}^{k}DE_{\text{mode}}\right]\;\;\text{for}\;G_{j}>0\\ \min\left[0,\;\;\gamma_{j}^{k}\left(G_{j}+\overset{\wedge }{\lambda }{\overset{\wedge }{\sigma }}^{2}\right)\right]=\min\;\left[0,\;\;\gamma_{j}^{k}DE_{\text{mode}}\right]\;\;\text{for}\;G_{j}<0 \end{array}$$

where ${\overset{\wedge }{\sigma }}^{2}=\frac{1}{n}{\overset{\wedge }{\sigma }}_{e}^{2}$, $G_{j}=\frac{1}{n}{\text{b}}_{j}{}^{\prime }\;{\text{y}}_{-j}=\frac{1}{n}\left({\text{b}}_{j}{}^{\prime }\;\text{y}-\sum_{l\neq j}{\text{b}}_{j}{}^{\prime }{\text{b}}_{l}\;{\overset{\wedge }{g}}_{l}^{k}\right)$ and ${\text{y}}_{-j}=\text{y}-\sum_{l\neq j}{\text{b}}_{l}\;{\overset{\wedge }{g}}_{l}^{k}$. As shown in Additional file 2, *G*_*j *_is the maximum likelihood (cML) estimate of *g*_*j *_conditional on all other SNP estimates. Hence ${\overset{\wedge }{g}}_{j}$ is a proportion of the cML estimate (*G*_*j*_) after shrinking it toward 0 due to the DE prior for *g*_*j*_. If *γ *= *1 *then ${\overset{\wedge }{g}}_{j}$ is the LASSO estimate of *g*_*j *_as the posterior mode for a DE prior is the LASSO [10,11]. However only a proportion of this Bayesian posterior mode is used due to the effect of the Dirac Delta prior where the proportion used is the posterior probability that the SNP is in LD with QTL. In fact as shown in Additional file 2${\overset{\wedge }{g}}_{j}$ can be written as a weighted mean of two posterior modes; one is the posterior mode when the DE is the only prior (*DE*_mode_) and the other is the posterior mode when the Dirac Delta is the only prior (*DD*_mode_). That is,

$${\overset{\wedge }{g}}_{j}=\gamma_{j}^{k}DE_{\text{mode}}+\left(1-\gamma_{j}^{k}\right)DD_{\text{mode}}=\gamma_{j}^{k}DE_{\text{mode}}$$

as the *DD*_mode _is always zero (i.e. *DD*_mode _= *0*) reflecting the posterior certainty due to the Dirac Delta prior certainty about the SNP effect.

It is also shown in equation (B9) of Additional file 2 that the ML estimators of *γ, λ *and $\sigma_{e}^{2}$ are as follows:

(8) $$\overset{\wedge }{\gamma }=\frac{1}{m}1^{\prime }\gamma^{\text{k}},\overset{\wedge }{\lambda }=\frac{1^{\prime }\gamma^{\text{k}}}{\gamma^{\text{k}}{}^{\prime }\left|\overset{\wedge }{\text{g}}\right|}\text{ and }{\overset{\wedge }{\sigma }}_{e}^{2}=\frac{1}{n}{\left(\text{y}-B\overset{\wedge }{\text{g}}\right)}^{\prime }\left(\text{y}-B\overset{\wedge }{\text{g}}\right)$$

where **γ**^**k **^is the vector of posterior probabilities at iteration *k*.

### emBayesB using Gauss-Seidel iteration

The steps in the EM algorithm using Gauss-Seidel (GS) iteration are as follows:

*Step 1*. Start with an initial set of values for ${\overset{\wedge }{g}}_{j},\overset{\wedge }{y},\;\overset{\wedge }{\lambda }$ and ${\overset{\wedge }{\sigma }}_{e}^{2}$. For example, ${\overset{\wedge }{g}}_{j}$ = 0, $\overset{\wedge }{\gamma }=0.01$, (or similar guessed value), ${\overset{\wedge }{\sigma }}_{e}^{2}=\left(1-h^{2}\right)\sigma_{y}^{2}$ (for a guessed heritability *h*^2^), and λ∧=(2mγ∧/h2σy2)12 as the variance of a DE is *2/λ*^*2 *^and so the total genetic variance is ${h^{2}\sigma_{y}^{2}=2m\gamma /\lambda }^{2}$.

*Step 2*. For SNP *j *(*j = 1,…,m*), calculate $G_{j}=\frac{1}{n}{\text{b}}_{j}{}^{\prime }\;{\text{y}}_{-j}=\frac{1}{n}\left({\text{b}}_{j}{}^{\prime }\;\text{y}-\sum_{l\neq j}{\text{b}}_{j}{}^{\prime }{\text{b}}_{l}\;{\overset{\wedge }{g}}_{l}^{k}\right)$ using Gauss-Seidel iteration and use these *G*_*j *_values to calculate the posterior probabilities $\gamma_{j}^{k}$ for iteration *k *as shown in equation (A4) of Additional file 1.

*Step 3*. Use the current estimates of $\gamma_{j}^{k}$ to update the MAP estimates of *g*_*j *_as shown in equation (7). Then update the ML estimates of *γ, λ *and $\sigma_{e}^{2}$ as shown in equation (8).

*Step 4*. Repeat *Steps 2 *and *3 *until convergence which is assessed at iteration *k *using the criterion ${\left({\overset{\wedge }{\text{g}}}^{k}-{\overset{\wedge }{\text{g}}}^{k-1}\right)}^{\prime }\left({\overset{\wedge }{\text{g}}}^{k}-{\overset{\wedge }{\text{g}}}^{k-1}\right)/\left({\overset{\wedge }{\text{g}}}^{k}{}^{\prime }{\overset{\wedge }{\text{g}}}^{k}\right)$. Small values of the criterion indicate that the estimates are not changing much relatively i.e. indicate convergence.

If needed, fixed effects can be fitted in the model simultaneously with the SNP effects as explained in [13].

### emBayesB for large SNP panels

In *Step 2 *of the EM algorithm using GS we calculate all possible combinations of **b**'_*j*_**b**_*l *_(i.e. **B**'**B**) each iteration. It is more computationally efficient to store the symmetric matrix **B**'**B **at the start. However this matrix is of order *m × m *which will be huge for large SNP panels. To avoid the calculation of **B**'**B **we use Gauss-Seidel iteration with residual update (GSRU) as described in [16] where it was used to avoid the calculation of **B**'**B **in a heuristic BLUP approach to genomic selection. Basically GSRU avoids the calculation of **B**'**B **by using the identity ${\text{y}}_{-j}=\text{y}-\sum_{l<j}{\text{b}}_{l}\;{\overset{\wedge }{g}}_{l}^{k+1}-\sum_{l>j}{\text{b}}_{l}\;{\overset{\wedge }{g}}_{l}^{k}={\text{e}}^{k+1,j}+{\text{b}}_{j}\;{\overset{\wedge }{g}}_{j}^{k}$ where **e**^*k+1,j *^is the vector of estimated residuals at iteration *k + 1 *for the calculation of ${\overset{\wedge }{g}}_{j}^{k+1}$. Hence to implement Gauss-Seidel iteration with residual update (GSRU) *Steps 1 *and *4 *of the EM algorithm need no modification but *Steps 2 *and *3 *need to be changed. The new *Steps 2 *and *3 *are:

*Step 2*^*GSRU*^. For SNP *j *(*j = 1,…, m*), calculate $G_{j}=\frac{1}{n}{\text{b}}_{j}{}^{\prime }\;{\text{y}}_{-j}=\frac{{\text{b}}_{j}{}^{\prime }{\text{e}}^{k+1,j}}{n}+{\overset{\wedge }{g}}_{j}^{k}$ and use this *G*_*j *_value to calculate the posterior probability $\gamma_{j}^{k+1}$ for iteration *k + 1 *as shown in equation (A4) of Additional file 1. Then calculate ${\overset{\wedge }{g}}_{j}^{k+1}$ using equation (7) and immediately update **e **using ${\text{e}}^{k+1,j+1}={\text{e}}^{k+1,j}-{\text{b}}_{j}\left({\overset{\wedge }{g}}_{j}^{k+1}-{\overset{\wedge }{g}}_{j}^{k}\right)$ before the calculation of *G*_*j+1*_. The update of **e **results from the identity ${\text{y}}_{-j}={\text{e}}^{k+1,j}+{\text{b}}_{j}\;{\overset{\wedge }{g}}_{j}^{k}={\text{e}}^{k+1,j+1}+{\text{b}}_{j}\;{\overset{\wedge }{g}}_{j}^{k+1}$ which links the two estimates of *g*_*j*_.

*Step 3*^*GSRU*^. Now update the ML estimates of *γ, λ *and $\sigma_{e}^{2}$ using equation (8).

As mentioned in [16], **e **should be recalculated periodically (e.g. at each iteration) using ${\text{e}}^{k+1}=\text{y}-\text{B}{\overset{\wedge }{\text{g}}}^{k}$ as numerical errors can accumulate in the procedure suggested for updating **e**.

### Simulation example

To benchmark the capabilities of emBayesB the SNP data distributed to participants of the QTLMAS XII workshop was analysed. A summary of the data simulation is given here, with full details available in [17]. An initial population of 50 male and 50 female founder individuals was created. For the next 50 generations, 50 males and 50 females were produced by random sampling parents each generation. For the last six generations, 15 males and 150 females were selected randomly for a hierarchical mating, with each male mated randomly to 10 females who produce 10 progeny each, giving a total of 1500 pedigreed progeny per generation. The 1200 individuals in the validation data set consisted of a random sample of 400 progeny from each of the last three generations. The 4665 individuals in the training data set were progeny from the preceding four generations; three generations of 1500 progeny plus the initial 15 males and 150 females. The training data set contained both SNP genotypes and phenotypic records, while the validation data contained only SNP genotypes.

**Figure 1:**
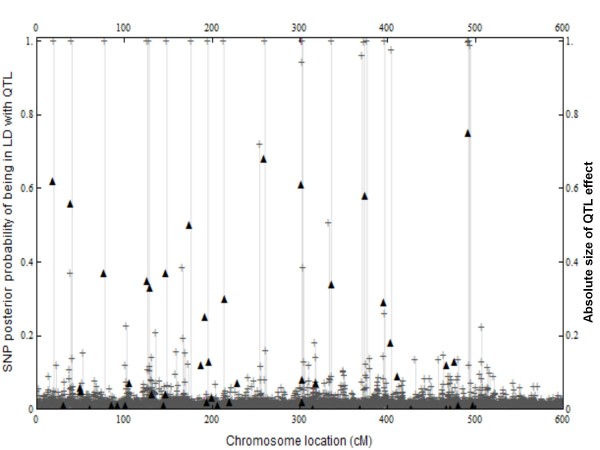
**48 QTL effects and 5726 SNP posterior probabilities of being in LD with QTL**. Average effect of an allelic substitution in the training data set (▲) plotted against genomic location for each of the 48 QTL. Also the SNP posterior probability (**+**) of being in LD with at least one QTL plotted against genomic location for each of the 5726 SNP. The QTL effects are in absolute values.

There were 6000 biallelic marker loci on six 100 cM chromosomes with a 0.1 cM spacing between marker loci which gave 1000 markers per chromosome. Marker alleles were sampled with equal probability in the founders. QTL effects were sampled from a gamma distribution. The genomic location and allele substitution effects of the 48 simulated biallelic and additive QTL are shown in Figure 1. More detail about the QTL effects is available in [18]. The number of QTL which explain more than 0.1, 1, 5 and 10% of the total genetic variation in the training data set were 28, 15, 6 and 4 respectively. The true breeding value (TBV) of an individual was calculated as the sum of its QTL effects. Phenotypic records were calculated for the training data set by adding a normally distributed residual error term to each individual's TBV. The variance of the normally distributed residual error term was chosen to produce a heritability of 0.3 for the trait.

#### Statistical analysis

The prediction equation $\text{GEBV}=\text{B}\overset{\wedge }{\text{g}}$ was determined for emBayesB using GS iteration by analysing the phenotypes and SNP genotypes of the 4665 individuals in the training data set. The number of SNP analysed was 5726 as only SNP with a minor allele frequency greater than 0.05 were used. The initial parameter estimates assumed for emBayesB were ${\overset{\wedge }{g}}_{j}$ = 0, $\overset{\wedge }{\gamma }=0.01$, plus $\overset{\wedge }{\lambda }$ and ${\overset{\wedge }{\sigma }}_{e}^{2}$ for an observed phenotypic variance of 4.42 and heritabilities of 0.1, 0.3, 0.5, 0.7 and 0.9. The algorithm was stopped when the convergence criteria was less than *1 × 10*^*-8*^. The prediction equation was used to calculate the GEBV of the 1200 individuals in the validation data set using only the genotype of their 5726 SNP. The accuracy of the prediction equation was determined by correlating GEBV and TBV separately for each of the 3 generations (400 individuals) of the validation data and combined over all 3 generations. The linear regression of TBV on GEBV was also calculated as a slope of one indicates that the GEBV are unbiased. Spearman's rank correlation was calculated for the top 10% of individuals ranked on TBV in the validation data.

GEBV were also calculated for GS-BLUP, LASSO and the ICE algorithm. The estimated SNP effects for GS-BLUP were solutions to $\left({\text{B}}^{\prime }\text{B}+\alpha \;Ι\right)\overset{\wedge }{\text{g}}={\text{B}}^{\prime }\text{y}$, where $\alpha =\sigma_{e}^{2}/\sigma_{g}^{2}=m\left(1-h^{2}\right)/h^{2}$. LASSO estimates where calculated using emBayesB by fixing *γ *= *1 *in each analysis, and also by fixing *λ *and $\sigma_{e}^{2}$ at their initial values. Details of the ICE algorithm are given in [13]. ICE uses fixed values of *γ, λ *and $\sigma_{e}^{2}$. The Fortran 90 source code and Windows executable of the emBayesB algorithm (plus GS-BLUP, LASSO and ICE) can be found in Additional file 3.

The emBayesB algorithm had difficulty with estimation of *λ *for some heritabilties. This is probably a reflection of the flat likelihood surface for estimating *λ *particularly when combined with estimating *γ*. Hence an upper bound was placed on *λ *in each analysis with the upper bound being the corresponding *λ *used as the initial value for the LASSO. If the bound was reached then the current estimate of *λ *was reset to its initial value. This procedure seemed to produce a good searching algorithm for parameter estimation with emBayesB given the complexity of the likelihood surface.

## Results

### Comparison of methods using simulated data

The emBayesB algorithm, and indeed all methods in Table 1, took only a few minutes to converge on a 2 GHz laptop PC for the 6 k SNP panel simulated. This was considerably faster than a full Bayesian analysis similar to [6] which took approximately 2 days (R. Pong-Wong, pers. comm.). A similar difference in computer time was reported in [13] where ICE was compared with a full Bayesian analysis (an MCMC implementation of the BayesB algorithm).

**Table 1:** Correlation and regression coefficient of TBV on GEBV for various generations of the validation data.

			ICE		emBayesB		
			
**Gen**^***a***^	**h**^**2**^^***b***^	GS-BLUP	*γ *= *0.01*	*γ = 1*	**LASSO **$\gamma =1,\;\left(\lambda ,\;\sigma_{e}^{2}\right)$^***c***^	$\left(\gamma ,\;\lambda ,\;\sigma_{e}^{2}\right)$^***c***^	$\left(\gamma ,\;\lambda ,\;\sigma_{e}^{2}\right)$^***d***^
All^*e*^	0.1	0.75^*f *^(1.19)^*g*^	0.87 (0.89)	0.79 (1.22)	0.77 (1.49)	0.87 (0.89)	0.88 (1.13)
All	0.3	0.75 (0.85)	0.85 (0.86)	0.79 (0.92)	0.87 (1.15)	0.85 (0.86)	0.88 (1.05)
All	0.5	0.71 (0.69)	0.81 (0.79)	0.76 (0.78)	0.87 (1.00)	0.80 (0.78)	0.88 (0.99)
All	0.7	0.66 (0.55)	0.74 (0.67)	0.72 (0.65)	0.77 (0.75)	0.74 (0.67)	0.87 (0.91)
All	0.9	0.57 (0.38)	0.58 (0.43)	0.55 (0.35)	0.57 (0.38)	0.58 (0.43)	0.87 (0.90)

1	0.5	0.74 (0.71)	0.84 (0.82)	0.78 (0.78)	0.87 (1.00)	0.84 (0.82)	0.88 (0.99)
2	0.5	0.73 (0.71)	0.81 (0.81)	0.78 (0.81)	0.87 (1.03)	0.80 (0.80)	0.88 (1.02)
3	0.5	0.68 (0.62)	0.77 (0.71)	0.74 (0.72)	0.85 (0.92)	0.77 (0.70)	0.86 (0.92)

Correlation between TBV and GEBV, and the regression coefficient (in brackets) of TBV on GEBV for each generation, and for all three generations combined, of the validation data for GS-BLUP, ICE and emBayesB. Unless indicated otherwise, the initial parameter estimates are *γ *= *0.01*, $\sigma_{e}^{2}=\left(1-h^{2}\right)\sigma_{y}^{2}$ and λ=(2mγ/h2σy2)12 where $\sigma_{y}^{2}=4.42$ is the total phenotypic variance in the training data. The true heritability was 0.3

^*a *^Generations of the validation data used.

^*b *^Initial heritability assumed.

^*c *^Parameters in brackets are fixed at the initial values.

^*d *^Parameters in brackets are all estimated.

^*e *^All three generations combined.

^*f *^Correlation between TBV and GEBV.

^*g *^Regression coefficient (in brackets) of TBV on GEBV.

emBayesB was the most accurate method of predicting TBV in the validation data over all heritabilities (Table 1). The emBayesB correlation of 0.88 between GEBV and TBV for all 1200 individuals was similar to correlations of 0.84 to 0.87 for Bayesian MCMC methods performed on the same data, but larger than correlations of 0.5 to 0.77 for various BLUP models [17]. GS-BLUP produced correlations of 0.75, 0.71 and 0.66 for heritabilities 0.3, 0.5 and 0.7 respectively (Table 1). Using the top 10% of individuals ranked on TBV in the validation data, the calculated Pearson correlation was 0.51 for emBayesB, while the rank correlation between GEBV and TBV was 0.41 when initial heritability was 0.5. This rank correlation was lower than the rank correlations of 0.46 to 0.58 for Bayesian MCMC methods applied to the same data [17] but larger than the GS-BLUP rank correlation of 0.27.

ICE with *γ *= *0.01 *produced a correlation of 0.87 when heritability was 0.1 (Table 1). However the correlations for ICE decreased as initial heritability increased, whereas for emBayesB the correlations remained constant due to the ability of the EM algorithm to estimate the unknown parameters. If the emBayesB parameters γ, *λ *and $\sigma_{e}^{2}$ were fixed at their initial values then the correlations for emBayesB were practically identical to those for ICE (Table 1). Predicting TBV separately for each generation it was found that the accuracy for both ICE and GS-BLUP decreased considerably by the 3^rd ^generation whereas the accuracy for emBayesB decreased very little over generations (Table 1).

The LASSO produced similar correlations to emBayesB when heritability was 0.3 and 0.5, but smaller correlations when heritability was 0.1, 0.7 and 0.9 (Table 1). Heritabilities of 0.1, 0.3, 0.5, 0.7 and 0.9 correspond to *λ *values of 161, 93, 72, 61 and 54 respectively for the LASSO. As *λ *decreases the LASSO performs less shrinkage such that the number of non-zero LASSO estimates of SNP effects increases and was 20, 57, 132, 233 and 1029 as heritability increased in Table 1. In practice the *λ *value would usually be determined by cross validation for the LASSO. When heritability was 0.3 or 0.5, the LASSO correlations decreased very little across the three generations similar to emBayesB (Table 1). Using *γ *= *1 *the ICE algorithm was not able to match the performance of the LASSO which used a fixed *γ *= *1 *in the emBayesB algorithm with all other parameters fixed (Table 1). The reason for this result is illustrated graphically later.

The regression of TBV on GEBV was biased for GS-BLUP and ICE for all heritabilities in Table 1. For emBayesB and the LASSO the regression of TBV on GEBV was only unbiased when heritability was 0.5 although emBayesB displayed the least bias for each heritability.

For an initial heritability of 0.5, the final parameter estimates were $\overset{\wedge }{\gamma }\;=0.023$, $\overset{\wedge }{\lambda }=31.41$ and ${\overset{\wedge }{\sigma }}_{e}^{2}=3.08$ for emBayesB. If we assume the number of SNP in LD with QTL is 48, then the true parameters are *γ *= 48/5726 = 0.0084, λ=(2mγ/h2σy2)12=((2×48)/(0.3×4.42))12=8.51 and $\sigma_{e}^{2}=\left(1-h^{2}\right)\sigma_{y}^{2}=3.09$. The estimated genetic variance ${\overset{\wedge }{\sigma }}_{a}^{2}={2m\overset{\wedge }{\gamma }/\overset{\wedge }{\lambda }}^{2}=0.264$ was an underestimate of the true genetic variance $\sigma_{a}^{2}=h^{2}\sigma_{y}^{2}=1.33$. These estimates produced a heritability of 0.08 whereas the true heritability was 0.3. This underestimation is not surprising given the incomplete LD between SNP and QTL. This helps explain why ICE produced its largest correlation between TBV and GEBV for a heritability of 0.1 in Table 1.

### SNP results for emBayesB when *h^2 ^*= *0.5*

The SNP results that follow were obtained using emBayesB with an initial heritability of 0.5. As expected most SNP have a small posterior probability of being in LD with at least one QTL (Figure 1). In fact 5660 SNP have posterior probabilities less than 0.1, while only 27 SNP have posterior probabilities greater than 0.5. emBayesB detected all QTL with allele substitution effects greater than 0.18 by calculating posterior probabilities of 0.98 or more for nearby SNP (Figure 1). On chromosome 6 all SNP have posterior probabilities less than 0.22 which was in accord with the absence of QTL simulated on this chromosome.

**Figure 2:**
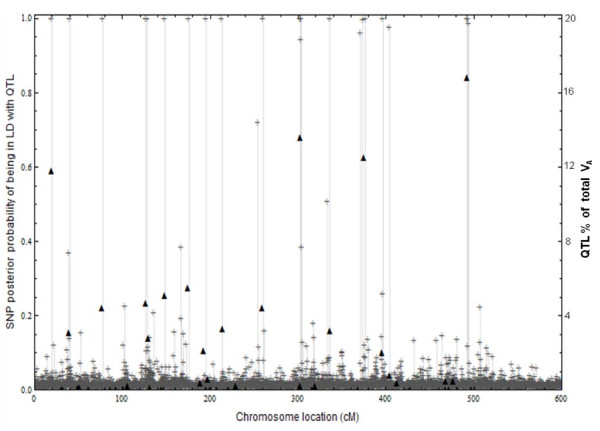
**Genetic variation explained by each of the 48 QTL and 5726 SNP posterior probabilities**. Percentage of the total genetic variance in the training data set explained by each QTL (▲) plotted against genomic location for each of the 48 QTL. Also the SNP posterior probability (**+**) of being in LD with at least one QTL plotted against genomic location for each of the 5726 SNP.

Of the 48 QTL simulated, there were 15 QTL which, individually explained more than 1% of the total additive genetic variation, and in total, explained over 95% of the additive genetic variance. emBayesB detected each of these 15 QTL by calculating posterior probabilities of 0.99 or more for nearby SNP (Figure 2). The distance from each of these 15 QTL to the nearest high probability SNP averaged 0.7 cM, with the largest distance being 1.7 cM. Three QTL each explained more than 12% of the genetic variation and this large variation resulted in multiple nearby SNP having posterior probabilities of 1 (Figure 2).

There were 25 SNP with posterior probabilities greater than 0.9 and the distance averaged 0.85 cM from each of these 25 SNP to the nearest QTL explaining more than 1% of the total genetic variation. As the genetic variation explained by a QTL dropped below 1%, the posterior probability of nearby SNP decreased toward zero. Hence in this simulation it was found that the SNP posterior probabilities could be used to accurately locate QTL explaining more than 1% of the total genetic variation.

In general the SNP used for prediction were different for emBayesB and the LASSO. For example with an initial heritability of 0.5, the number of estimated SNP effects greater than 0, 0.01 and 0.1 was 2841, 15 and 10 for emBayesB compared with 132, 72 and 6 for the LASSO. However the LASSO did use SNP which emBayesB estimated as having a non-zero posterior probability of being in LD with QTL. For example, the LASSO used 57 and 132 non-zero estimates of SNP effects for heritabilities of 0.3 and 0.5 respectively, and these SNP had average posterior probabilities of 0.31 and 0.16 of being in LD with QTL as estimated by emBayesB.

### Analytical emBayesB shrinkage

**Figure 3:**
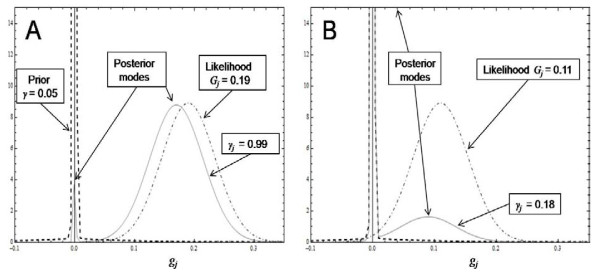
**Graphical illustration of how a posterior probability is calculated for a SNP**. Graphs of the mixture prior *p*(*g*_*j*_), conditional likelihood *h*(*g*_*j*_|*G*_*j*_, *σ*^*2*^) and conditional posterior distribution *p*(*g*_*j *_|**g**_*-j,*_**y**) as given in equations (A1) and (A2) of Additional file 1 for $\gamma =0.05,\;\lambda =10,\;\sigma_{e}^{2}=1$ and *n = 500*. The Dirac Delta function is replaced by a DE with *λ*_*s *_= *1000 *i.e. a Spike at 0. The posterior probability *γ*_*j *_of SNP *j *being in LD with QTL is calculated from equation (A3) by numerical integration. Figures A and B show the distributions when *G*_*j *_is 0.19 and 0.11 respectively, where *G*_*j *_is the conditional maximum likelihood estimate of *g*_*j*_.

In this section we graphically explore features of emBayesB in order to assist with understanding how the algorithm works. Figure 3 shows the shape of the conditional posterior distribution of *g*_*j *_given in equation (A2) of Additional file 1. The graphs assume $\gamma =0.05,\;\lambda =10,\;\sigma_{e}^{2}=1$ and *n = 500 *plus we have used a DE with *λ*_*s *_= 1000 (i.e. a Spike at 0) to replace the Dirac Delta function as done in Additional file 2. The mixture prior in Figure 3 is given in equation (A1) of Additional file 1. We call the function *h*(*g*_*j*_|*G*_*j*_, *σ*^2^) in Figure 3 a Likelihood as it is the normally distributed conditional likelihood derived in Appendix 2 of [13].

When the cML estimate (*G*_*j*_) of *g*_*j *_is far from 0 the conditional posterior resembles the conditional likelihood, but is slightly shifted (or shrunk) toward 0. The mode of the shrunk likelihood is *G*_*j*_-*λσ*^*2*^(=*G*_*j*_-*0.02*) when *G*_*j *_is much greater than 0. This is the LASSO estimate as the Spike has little influence when *G*_*j *_is far from 0. However as *G*_*j *_approaches 0, the conditional posterior becomes bimodal, with the height of the mode at 0 increasing the closer *G*_*j *_is to 0 (Figure 3). This reflects the fact that, the closer *G*_*j *_is to 0, the higher is the probability that the true *g*_*j *_is 0. Using numerical integration in equation (A3) it can be shown that the area under the DE part of the conditional posterior is 0.99, 0.67 and 0.18 for *G*_*j *_values of 0.19, 0.15 and 0.11 as shown in Figure 3. In the EM algorithm this DE area is $\gamma_{j}^{k}$, the posterior probability that SNP *j *is in LD with at least one QTL given the data and all other current estimates at iteration *k*.

Using numerical integration it can also be shown that the mean of the conditional posterior is 0.1677, 0.0868 and 0.0165 for *G*_*j *_values of 0.19, 0.15 and 0.11 respectively, while the MAP estimates of *g*_*j *_(calculated using equation (7)) are 0.1677, 0.0868 and 0.0163 for the same values of *G*_*j*_. So the MAP estimate of *g*_*j *_is an accurate estimate of the conditional posterior mean. Hence at convergence, it is reasonable to expect that the MAP estimate will be an accurate estimate of the marginal posterior mean of *g*_*j*_. Bayesian MCMC methods use the marginal posterior mean of each SNP in the prediction equation $\text{GEBV}=\text{B}\overset{\wedge }{\text{g}}$, whereas emBayesB uses the MAP estimate given in equation (7). Hence it is not surprising to find that emBayesB has a similar accuracy of prediction compared to Bayesian MCMC methods as found in the analysis of the simulated workshop data.

**Figure 4:**
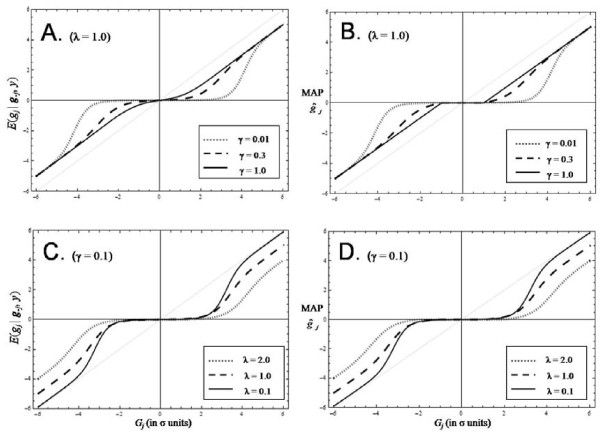
**Shrinkage of the cML estimate using the posterior mean or the MAP estimate**. Plots of the analytical formulae for the conditional posterior mean *E*(*g*_*j*_*|****g***_*-j*_,**y**) (Figures 4A and 4C) as given in Appendix 1 of [13] and the MAP estimate of *g*_*j *_(Figures 4B and 4D) as given by equation (7) with the posterior probability *γ*_*j *_given by equation (A4). All plots have *G*_*j *_(the conditional ML estimate of *g*_*j*_) on the horizontal axis. *G*_*j *_is in *σ *units as all plots use *σ = 1*.

The analytical relationship between the conditional posterior mean *E*(*g*_*j *_| **g**_*-j*_,**y**) and the MAP estimate of *g*_*j *_is explored further in Figure 4. The analytical derivation of *E*(*g*_*j *_| **g**_*-j*_,**y**) is given in Appendix 1 of [13], while the MAP estimate of *g*_*j *_is calculated using equation (7) with *γ*_*j *_given by equation (A4). Plots of the *E*(*g*_*j *_| **g**_*-j*_,**y**) versus *G*_*j *_are given in Figures 4A and 4C, while plots of the MAP estimate ${\overset{\wedge }{g}}_{j}$ versus *G*_*j *_are given in Figures 4B and 4D. The most striking feature is the similarity of the paired curves when comparing Figures 4A and 4B (*λ *= *1.0 *and the same *γ*), or when comparing Figures 4C and 4D (the same *λ *and *γ = 0.1*). Once again it seems that the MAP estimate of *g*_*j *_is an accurate estimate of the conditional posterior mean as found earlier. The difference between the paired curves is largest when *γ = 1 *and *G*_*j *_is close to 0 as can be seen in Figures 4A and 4B.

When *γ = 1 *in Figure 4B, the MAP curve resembles a broken stick which is absolutely flat around the origin. This is the LASSO estimate which is a broken stick for all values of *λ*. The LASSO's ${\overset{\wedge }{g}}_{j}$ estimate is shrunk the constant amount of *λσ*^*2 *^(= 1 in Figure 4B) from the cML estimate of *G*_*j *_as shown in equation (7) when *G*_*j *_is past the break in the stick. As the value of *γ *decreases in Figure 4B, the asymptotic value of *G*_*j*_±*λσ*^*2*^(=*DE*_mode_) is shrunk even more, and in a non-linear manner, as *G*_*j *_approaches the origin, with greater shrinkage for smaller *γ *values. This is due to the *a priori *belief that a proportion (*1 -γ*) of the SNP are 0 and so small values of *G*_*j *_are more probably 0, and so shrunk more, as *γ *decreases. In fact the shrinkage is proportional to *γ*_*j *_as shown in equation (7).

When *γ = 0.1*, the MAP curves show strong shrinkage to 0 for any *G*_*j *_values between *-2σ *and *2σ *for all values of *λ *(Figure 4D). Even more shrinkage of small *G*_*j *_values occurs when *γ = 0.01 *as the *G*_*j *_interval increases to (*-3σ*, *3σ*) as shown in Figure 4B. As *λ *increases in Figure 4D, the variance of the DE distribution gets smaller which results in a smaller total genetic variance for fixed *m *and *γ*. Hence we need more shrinkage (±*λσ*^*2*^) of large |*G*_*j*_| values as shown by the different asymptotes in Figure 4D.

## Discussion

This study has developed a fast EM algorithm for genome wide prediction in which there is a joint prediction of breeding value from accumulated SNP data. The benefits of the algorithm are its fast performance, its verity in relation to the proposed model, and the optimality properties it brings from application of the EM algorithm. The time advantage of emBayesB over a full Bayesian analysis is expected to be even greater with dense 500 k SNP panels currently being used in GWA studies. A disadvantage of emBayesB is that no standard errors are routinely available from an EM algorithm. However there are methods of obtaining standard errors with an EM algorithm [14] and even bootstrapping is a possibility given the fast performance.

The predictive power of emBayesB comes from the use of the information-rich prior mixture distribution which is of particular value when the number of QTL is small relative to the number of independent genomic segments [19]. In fact it is expected that there will be no advantage in using emBayesB over GS-BLUP if the simulated QTL more closely fit an infinitesimal model. As with other recent studies [10,11] emBayesB uses a DE prior distribution for QTL effects which has some experimental justification [8]. In addition emBayesB incorporates *a priori *that an unknown proportion of SNP will not be in LD with QTL through the use of the Dirac Delta function in the prior mixture distribution for the SNP effects. This SNP prior mixture is quite different to that used in the EM algorithm wBSR in [15] where the Dirac Delta function was not used to model the absence of LD. The wBSR algorithm derived in [15] is only an approximate EM algorithm due to the approximation used to include the missing data variable *γ*_*l *_(the SNP weight) into the EM modelling process. Using a Dirac Delta function in the prior mixture seems a more theoretically attractive way of modelling the LD between SNP and QTL and produces some appealing analytical results like the posterior probability formula in equation (A4) and the result that the best estimate of a SNP effect can be viewed as a regressed DE mode as shown in equation (7).

emBayesB is an EM algorithm which has similarities with the fast heuristic algorithm called ICE [13]. ICE uses the same formulation of the data model and the SNP prior distribution but iterates on the mean of each SNP effect conditional on all the other SNP mean effects, the *y *data and assuming fixed values for *γ, λ *and $\sigma_{e}^{2}$. It is unknown in general how optimal ICE solutions are. But if the fixed values of *γ, λ *and $\sigma_{e}^{2}$ assumed in ICE are set equal to the ML estimates obtained from emBayesB then we have found that the prediction accuracy of ICE is identical to the prediction accuracy of emBayesB (e.g. see *h*^*2 *^= *0.1 *in Table 1). This seems to reinforce the conclusion drawn from Figure 4 that the posterior mean of a SNP effect is well approximated by the MAP estimate in equation (7). Hence it is no surprise to find that the accuracy of prediction calculated in the simulated example of [13] was similar for ICE and a Bayesian MCMC implementation of the BayesB model as ICE assumed fixed values of *γ, λ *and $\sigma_{e}^{2}$ which were close to optimal.

The simulated example of [13] used an 8010 SNP panel with 1000 individuals in the training and validation data sets. The speed advantage of ICE was large; ICE converged in 2 to 5 minutes compared to 47 hours for the Bayesian MCMC analysis. The computational speed advantage of ICE comes from the analytical calculation of the conditional posterior mean; emBayesB uses a similar analytical calculation for the conditional posterior probability. As ICE and emBayesB took similar amounts of CPU time in Table 1, the results for ICE in [13] provide further evidence of the computational speed advantage of emBayesB over a full Bayesian analysis.

emBayesB is similar to the empirical Bayes method suggested by [20] where Bayesian hyperparameters are estimated by marginal and conditional maximum likelihood methods. Taking an empirical Bayes approach in a wavelet regression application, [21] used marginal maximum likelihood with various prior mixtures involving the Dirac Delta function (including the DE as in emBayesB) to evaluate shrinkage of wavelet noise. They compared the posterior mean and posterior median as shrinkage methods and showed that the posterior median, unlike the posterior mean, produces a threshold rule for estimation in that estimated wavelet coefficients below a calculated threshold were set to exactly zero. The emBayesB estimate of a SNP effect is also calculated using a thresholding rule (see equation (7) and Figure 4). As with emBayesB, the empirical Bayes methods of [21] combine fast computation with good theoretical properties.

The simulated example used in this paper did not show any advantage for emBayesB over the LASSO. However in a simulated example of wavelet denoising, [22] demonstrated an advantage over the LASSO of both a Bayesian sigmoid model and the empirical Bayes method of [21] which uses a DD+DE mixture prior like in emBayesB. In fact various methods for shrinking coefficients in regression models were compared in [22] including the Bayesian sigmoid model which has a single hyperparameter to tune the shrinkage. The bimodal nature of the marginal posterior for a regression coefficient in the Bayesian sigmoid model (Figure 2 in [22]) is very similar to the bimodal nature of the conditional posterior distribution of a SNP effect as shown in Figure 3. The shape of the shrinkage graph for the Bayesian sigmoid model (Figure 4 in [22]) is also similar to the emBayesB shrinkage graph when *γ *is small and *λ *is small (see Figure 4D with *γ = 0.1, λ = 0.1*). However emBayesB will estimate values for *γ *and *λ*, and so, unlike the Bayesian sigmoid model, emBayesB can adapt its shrinkage such that it is appropriate for the prevailing nature of the data like in Figure 4.

## Conclusions

This paper reports an EM algorithm called emBayesB for genome wide prediction in which there is a joint prediction of breeding value from dense SNP marker data. A formulation of the emBayesB algorithm using GSRU is developed to handle large SNP panels. Using a simulated and widely available dataset it was found that the accuracy of emBayesB was similar to Bayesian approaches, but emBayesB took only a fraction of the computational time. Using emBayesB may be a promising solution to the problem found in GWA studies with the use of stringent statistical thresholds. The emBayesB calculation of posterior probabilities of SNP being in LD with QTL may also be useful in the area of SNP subset selection. Due to the fast computational speed, opportunities exist with emBayesB to explore fitting innovative models which could include non-additive genetic variation or even simultaneous fitting of multiple traits. More research is needed to explore the opportunities which emBayesB offers and to benchmark its capabilities.

## Availability and requirements

The simulated data analysed in the paper is available on the 12^th ^QTLMAS workshop website http://www.computationalgenetics.se/QTLMAS08/QTLMAS/DATA.html. The program emBayesB is available both as Fortran 90 source code and as a Windows executable in Additional file 3.

## Authors' contributions

RKS developed the emBayesB theory, wrote the emBayesB software and the paper. JAW developed aspects of the emBayesB theory and wrote sections of the paper. THEM formulated the basic Gauss Seidel algorithm, and helped formulate the research and writing the paper. All authors read and approved the final version of the paper.

## Supplementary Material

Additional file 1 **Appendix A**. A pdf file giving the E-step of the EM algorithm: Derivation of $\gamma_{j}^{k}$ the posterior probability that SNP *j *is in LD with at least one QTL. Click here for file

Additional file 2 **Appendix B**. A pdf file giving the derivation of the estimators ${\overset{\wedge }{g}}_{j},\overset{\wedge }{y},\;\overset{\wedge }{\lambda }$ and ${\overset{\wedge }{\sigma }}_{e}^{2}$ for the M-step of the EM algorithm. Click here for file

Additional file 3 **emBayesB.zip**. The zip file "emBayesB.zip" contains the Fortran 90 source code "emBayesB_gs.f90" and the Windows executable "emBayesB_gs.exe" for the emBayesB program. A readme file gives instructions on using the program and the input/output files. The two input data files "emBayesB_input*.txt" are also in the zip file. Click here for file
