# Supplementary material for: Genomic selection and complex trait prediction using a fast EM algorithm applied to genome-wide markers
Source: BMC Bioinformatics. 2010 Oct 22;11:529. doi: 10.1186/1471-2105-11-529 (PMC3098088; doi:10.1186/1471-2105-11-529)
Supplement: Additional file 1 — Appendix A. A pdf file giving the E-step of the EM algorithm: Derivation of γjk the posterior probability that SNP j is in LD with at least one QTL. [file 1471-2105-11-529-S1.DOC]

# Appendix A

**Derivation of $\gamma_{j}^{k}$ the posterior probability that SNP *j* is in LD with QTL**

To determine $\gamma_{j}^{k}\left(=E\left[z_{j}|\hat{\mathbf{g}}^{k},\mathbf{y}\right]\right)$ we need to calculate the probability of SNP *j* being in LD with QTL given the data and all current estimates at iteration *k*. That is, the probability that $g_{j}^{k}$ is from the double exponential part of the conditional posterior distribution for $g_{j}^{k}$. To do this we need to construct $p(g_{j}|\mathbf{g}_{-j}^{k},\mathbf{y})$ the conditional posterior distribution for $g_{j}^{k}$ given the data **y** and $\hat{\mathbf{g}}_{-j}^{k}$ which is the vector of all current SNP estimates except SNP *j*. We proceed as follows.

The prior distribution for SNP effect $g_{j}$ can be written as the mixture

(A1)

and can be used to construct the following formulation of the posterior for **g** given **y**

where $f\left(\mathbf{y}| \mathbf{g}\right)$ is given in equation (5). Using this full posterior we can show that the conditional posterior distribution for $g_{j}$ is

(A2)

where $G_{j}$ and $\sigma^{2}$ are defined in equation (7), $h\left(g_{j}|G_{j},\sigma^{2}\right)=\text{exp}\left(\frac{\left(g_{j}-G_{j}\right)}{2\sigma^{2}}\right)$ and K is the normalising constant. The derivation of $h\left(g_{j}|G_{j},\sigma^{2}\right)$ uses the same argument as used in Appendix 2 of [13]. Now the posterior probability $\gamma_{j}^{k}$ is as follows.

(A3)

A similar expression to the numerator of equation (A3) is evaluated in Appendix 1 of [13]. So using a similar derivation it can be shown that

$\gamma_{j}^{k}=\frac{DE_{part}}{DE_{part}+DD_{part}}$ (A4)

where $DE_{part}=\exp\left(-\lambda G_{j}\right)\left[1-\Phi\left(0;G_{j}^{-},\sigma^{2}\right)\right]+\exp\left(\lambda G_{j}\right)\Phi\left(0;G_{j}^{+},\sigma^{2}\right)$,

, $G_{j}^{+}=G_{j}+\lambda\sigma^{2}$, $G_{j}^{-}=G_{j}-\lambda\sigma^{2}$, and $\Phi\left(t;\mu,\sigma^{2}\right)=P\left(Z<\frac{t-\mu}{\sigma}\right)$ for $Z\sim N(0,1 )$.
