# Supplementary material for: Genomic selection and complex trait prediction using a fast EM algorithm applied to genome-wide markers
Source: BMC Bioinformatics. 2010 Oct 22;11:529. doi: 10.1186/1471-2105-11-529 (PMC3098088; doi:10.1186/1471-2105-11-529)
Supplement: Additional file 2 — Appendix B. A pdf file giving the derivation of the estimators g∧j,y∧, λ∧ and σ∧e2 for the M-step of the EM algorithm. [file 1471-2105-11-529-S2.DOC]

# Appendix B

**Derivation of $\hat{\text{g}}_{\text{j}}, \hat{\gamma}, \hat{\lambda}$ and $\hat{\sigma}_{\mathbf{e}}^{\mathbf{2}}$ for the M-step of the EM algorithm**

In the M-step we assume $\gamma_{j}^{k}$ is fixed and we maximize $E_{z}\left[\log p\left(\mathbf{z}, \mathbf{g}|\mathbf{y}\right)\right]$ for the unknown parameters $g_{j}, \gamma, \lambda$ and $\sigma_{e}^{2}$.

***Derivation of*** $\hat{\text{g}}_{\text{j}}$

As the Dirac Delta function doesn’t have a derivative we replace it in the prior by a DE with parameter $\lambda_{S}$ (to indicate a Spike at $g=0$ when $\lambda_{S}$ is very large). Later we let $\lambda_{S}\to\infty$ to derive $\hat{g}_{j}$ for the DD prior. The log posterior in equation (6) becomes

Using this log posterior we calculate $E_{z}\left[\log p\left(\mathbf{z}, \mathbf{g}|\mathbf{y}\right)\right]$ as follows

(B1)

Differentiating equation (B1) with respect to $g_{j}$ we obtain the following:

(B2)

where $sign\left(x\right)=1$ if $x>0$ and $sign\left(x\right)=-1$ if $x<0$. Setting this derivative to zero and rearranging, we obtain the following expression for $\hat{g}_{j}$

$\hat{g}_{j}=\left\{\begin{matrix} G_{j}-\gamma_{j}^{k}\hat{\lambda}\hat{\sigma}^{2}-\left(1-\gamma_{j}^{k}\right)\hat{\lambda}_{S}\hat{\sigma}^{2} \text{for} G_{j}>0 \\ G_{j}+\gamma_{j}^{k}\hat{\lambda}\hat{\sigma}^{2}+\left(1-\gamma_{j}^{k}\right)\hat{\lambda}_{S}\hat{\sigma}^{2} \text{for} G_{j}<0 \end{matrix}\right.$ (B3)

where $G_{j}$ and $\hat{\sigma}^{2}$ are defined in equation (7). So with no SNP prior ($\lambda=\lambda_{S}=0$) equation (B3) shows that $G_{j}$ is the maximum likelihood (cML) estimate of $g_{j}$ conditional on all other SNP cML estimates. Equation (B3) can be rewritten as

$\begin{matrix} \hat{g}_{j}=\left\{\begin{matrix} \gamma_{j}^{k}\left(G_{j}-\hat{\lambda}\hat{\sigma}^{2}\right)+\left(1-\gamma_{j}^{k}\right)\left(G_{j}-\hat{\lambda}_{S}\hat{\sigma}^{2}\right) \text{for} G_{j}>0 \\ \gamma_{j}^{k}\left(G_{j}+\hat{\lambda}\hat{\sigma}^{2}\right)+\left(1-\gamma_{j}^{k}\right)\left(G_{j}+\hat{\lambda}_{S}\hat{\sigma}^{2}\right) \text{for} G_{j}<0 \end{matrix}\right. \\ =\gamma_{j}^{k}DE_{\text{mode}}+\left(1-\gamma_{j}^{k}\right)Spike_{\text{mode}} \end{matrix}$ (B4)

where $DE_{\text{mode}}$ is the posterior mode when the DE with parameter $\lambda$ is the only prior, while $Spike_{\text{mode}}$ is the posterior mode when the DE with parameter $\lambda_{S}$ is the only prior. Now as $\lambda_{S}$ tends to infinity ($\lambda_{S}\to\infty$), we find that $Spike_{\text{mode}}\to DD_{\text{mode}}=0$ as the posterior mode ($DD_{\text{mode}}$) for a Dirac Delta prior is always zero. Hence $\hat{g}_{j}$ the estimate of SNP effect *j* can be calculated as follows

$\hat{g}_{j}=\gamma_{j}^{k}DE_{\text{mode}}=\left\{\begin{matrix} \max\left[0, \gamma_{j}^{k}\left(G_{j}-\hat{\lambda}\hat{\sigma}^{2}\right)\right] \text{for} G_{j}>0 \\ \min\left[0, \gamma_{j}^{k}\left(G_{j}+\hat{\lambda}\hat{\sigma}^{2}\right)\right] \text{for} G_{j}<0 \end{matrix}\right.$ (B5)

Equation (B5) includes the constraints that if $G_{j}>0$, the smallest value that $\hat{g}_{j}$ can be is zero and that if $G_{j}<0$, the largest value that $\hat{g}_{j}$ can be is zero.

***Derivation of* $\hat{\gamma}, \hat{\lambda}$ *and*** $\hat{\sigma}_{\mathbf{e}}^{\mathbf{2}}$

Differentiating equation (B1) with respect to $\gamma, \lambda$ and $\sigma_{e}^{2}$ we obtain the following:

$\frac{\partial}{\partial\gamma}\left\{E_{z}\left[\log p\left(\mathbf{z}, \mathbf{g}|\mathbf{y}\right)\right]\right\} =\frac{1}{\gamma}\sum_{j=1}^{m} \gamma_{j}^{k}+\frac{1}{\left(1-\gamma\right)}\sum_{j=1}^{m} \left(1-\gamma_{j}^{k}\right)$ (B6)

$\frac{\partial}{\partial\lambda}\left\{E_{z}\left[\log p\left(\mathbf{z}, \mathbf{g}|\mathbf{y}\right)\right]\right\} =\frac{1}{\lambda}\sum_{j=1}^{m} \gamma_{j}^{k}-\sum_{j=1}^{m} \gamma_{j}^{k}\left|g_{j}\right|$ (B7)

$\frac{\partial}{\partial\sigma_{e}^{2}}\left\{E_{z}\left[\log p\left(\mathbf{z}, \mathbf{g}|\mathbf{y}\right)\right]\right\} =-\frac{n}{2\sigma_{e}^{2}}+\frac{1}{2\left(\sigma_{e}^{2}\right)^{2}}\sum_{i=1}^{n} \left(y_{i}-\sum_{j=1}^{m} b_{ij} g_{j}\right)^{2}$ (B8)

Setting these derivatives to zero, we obtain the following formulae for $\hat{\gamma}, \hat{\lambda}$ and $\hat{\sigma}_{e}^{2}$

$\hat{\gamma} =\frac{1}{m}\sum_{j=1}^{m} \gamma_{j}^{k}=\frac{1}{m}\mathbf{1}'\mathbf{\gamma}^{\mathbf{k}}$, and $\hat{\sigma}_{e}^{2}=\frac{1}{n}\left(\mathbf{y-B}\hat{\mathbf{g}}\right)^{'}\left(\mathbf{y-B}\hat{\mathbf{g}}\right)$ (B9)

where $\mathbf{\gamma}^{\mathbf{k}}$ is the vector of SNP posterior probabilities at iteration *k*, $\mathbf{1}$ is a vector of ones, $\hat{\mathbf{g}}$ is the vector of SNP estimates $\hat{g}_{j}$ and $\left|\hat{\mathbf{g}}\right|$ is the vector of $\left|\hat{g}_{j}\right|$ values. Thus $\hat{\gamma}$ is the average posterior probability of a SNP being in LD with QTL. $\hat{\lambda}$ is the inverse of the weighted average of the absolute value of the estimated SNP effects. This result is not unexpected given that the ML estimator of $\lambda$ is $\hat{\lambda}=\frac{m}{\sum\left|X_{i}\right|}$ if the$X_{i}$ are a random sample of size *m* from a double exponential distribution. Another result which is not unexpected is that $\hat{\sigma}_{e}^{2}$ is the average of the squared residuals.
